# 3Shape Digital Design Software in Splints Creation—A Pilot Study

**DOI:** 10.1055/s-0041-1739546

**Published:** 2021-12-24

**Authors:** Dobromira Shopova, Miroslava Yordanova, Svetlana Yordanova

**Affiliations:** 1 Department of Prosthetic Dentistry, Faculty of Dental Medicine, Medical University, Plovdiv, Bulgaria; 2 Department of Orthodontics, Faculty of Dental Medicine, Medical University, Plovdiv, Bulgaria

**Keywords:** occlusal splints, bruxism, digital technologies, 3Shape

## Abstract

**Objectives** Digital technologies have widened their horizons into the world of dental medicine and now further expanding to cover all branches. This new modern technology replaces traditional laboratory techniques allowing effective patient care. Patients who suffer from bruxism—the act of involuntary habitual grinding of teeth—have widely been benefited by splint treatments. The aim of this article is to display the variety of occlusal splints that can be created by the 3Shape Digital Design Software and their application in specific clinical situations.

**Materials and Methods** Six variations in the splints were created digitally—three with uncombined designs and the remaining three with a combination of two of the main options available. During this study, 36 splints were made for patients aged 24 to 55 inclusively.

**Results** The largest number of splints according to the clinical picture were made of “raise to antagonist cusp tips” (14 pieces) and the remaining were of combined type “raise to antagonist cusp tips + raise to antagonist plane” (12 pieces). There thickness was within the range of 1.5 and 5 mm.

**Conclusion** 3Shape Digital Design Software—Splint Studio is a suitable system for designing and creating occlusal splints with respect to certain clinical situations. It is possible to combine the three main types in a separate section of the dental arch according to the case.

## Introduction

Bruxism is a common phenomenon within todays' society. Bruxism appears to be modulated by various neurotransmitters in the central nervous system. Factors such as smoking, alcohol, drugs, medical diseases, and trauma play a key role in the etiology of bruxism. Psychological factors, for example, stress and personality, are frequently mentioned as a key factor causing bruxism. 1 Studies have shown that individuals at high risk are individuals under daily stress such as students, career-driven individuals, individuals going through daily life troubles, and compassionate people. 2

Treatment of bruxism begins with the placement of an occlusal splint. In some cases, this is sufficient, but more often ongoing treatment is required, for example, fixing the new position by the methods of adhesive restorations, prosthetic structures, or orthodontic treatment. 3

Good quality splints have at least six functions, which are as follows (1) to relax the muscles, (2) to allow the condyle to seat in central relation, (3) to provide diagnostic information, (4) to protect teeth and associate structures from bruxism, (5) to mitigate the periodontal ligament proprioception, and (6) to reduce cellular hypoxia levels. 4 Restrepo et al observed that the use of rigid occlusal bite plates was not sufficient in reducing the signs of bruxism as a whole, but the splints were able to reduce the deviation in mouth opening. 5 Alkan et al suggested that occlusal splint therapy may be more effective than tricyclic antidepressant in the treatment of bruxism. 6

The digital work protocol significantly simplifies the technological process. It is possible for the dentist to design the splint alone or alongside a dental technician. This combines the knowledge of both specialties. The limits shown are not approximates but are 100% accurate. This accuracy is ensured as the software takes into account the error of material shrinkage therefore, limiting clinical adjustments. 7 8 Aly and Mohsen demonstrated that digital casts were known to show a significantly higher error than a stone cast; however, the errors indicated were within the acceptable clinical range. 9

The digital design allows the creation of different splints variants. Variations can be both with respect to boundaries and with respect to the type of occlusal surface. The boundaries are influenced by the retention area and positioning of the teeth. The occlusal surface of the splint is mainly affected by the type of occlusion, the type of occlusal surfaces (abraded or nonabraded), the loss of the vertical dimension of occlusion (VDO), temporo-mandibular joint disorders, and others. 10 11 Okeson summarized the findings of many different authors and considered that the two most commonly used splints are the stabilization appliance and the anterior positioning appliance. The stabilization appliance also known as a *muscle relaxation appliance* because of its primary use to reduce muscle pain. The anterior positioning appliance is also known as an *orthopedic repositioning appliance,* since its goal is to change the position of the mandible in relation to the cranium. Other types of occlusal devices are the anterior bite plane, the posterior bite plane, the pivoting appliance, and the soft or resilient appliance. The description of each appliance and the treatment goals for each is reviewed below alongside the indications for each. 12 13 14 The multitude of occlusal devices described in literature can be classified according to their indications that are as follows: (1) occlusal devices that are intended primarily to normalize muscle tone and improve neuromuscular coordination (relaxation splints, vertical dimension splints); (2) occlusal devices that serve primarily to reposition the mandible and decompress joint structures (repositioning splint, decompression splint, vertical dimension splint); (3) occlusal devices to stabilize the jaw relations that were established during the initial course of occlusal therapy, and to determine the most favorable centric and eccentric occlusal scheme for the final restorations (decompression splint, stabilization splint). 15 16

The therapeutic thickness of the splint has not yet been specified. Various authors agree that it is more correct to take into account the distance between the central incisors. The balanced splint is not of equal thickness in the frontal and distal sections. Pita et al observed no differences between the tested splint thicknesses (3 and 6 mm). Pita et al used an objective analysis conducted using electromyographic (EMG) evaluation and utilized an interocclusal separation (the greatest thickness was 6 mm). 17 EMG is an advanced technique to record and evaluate muscle activity. 18 In addition, Olthoff et al did not observe significant differences among the results of the chewing tests with or without splint and with 2, 4, or 6 mm increase in VDO.

This study used subjective criteria based only on symptoms reported by the patients, who reported absence of pain, discomfort, or tension in the masticatory muscles and no changes in masticatory performance. 19 In contrast, Suvinen et al reported a gradual reduction in the electrical activity of the masseter when the VDO was increased. The difference found between their study and ours can be attributed to the different VDOs that were used. These authors reported an interocclusal separation of ∼14 mm. 20 Occlusal relationships are present in each variation of the splint. 21 Infrared thermography imaging can be used to check muscle activity, and hence the effect of splint treatment. 22

The purpose of this article is to present a pilot study on the capabilities of 3Shape Digital Design Software—splint studio, with the main types of splints embedded software and the combinations between them.

## Materials and Methods

Digital software allows the creation of a variation in splints depending on the clinical picture. 3Shape Digital Design Software - splint design allows the creation of 3 main types of occlusal surface:

- Raise to antagonist cusp tips (RACT)
- Raise to antagonist plane (RAP)
- Raise ramp (RR)

These surfaces can be set in separate areas of the dentition and combined with each other. Most often, the same design is chosen for the distal areas and different for the frontal area. We chose the following combinations:

- RACT – distal + RAP – frontal
- RR – distal + RAP – frontal
- RAP – distal + RR – frontal

In the study, 36 patients aged between 24 and 55 years were involved. The number of patients and their distribution are illustrated in Table 1 .

**Table 1:** Distribution of splints by number of patients

	Types of splints	Number of patients
1.	Raise to antagonist cusp tips (RACT)	15
2.	Raise to antagonist plane (RAP)	3
3.	Raise ramp (RR)	2
4.	RACT – distal + RAP – frontal	12
5.	RR – distal + RAP – frontal	3
6.	RAP – distal + RR – frontal	1
	Total	36

Post completion of the design, all the splints were made by the method of 3D printing—biocompatible material *Dental LT Clear Resin* —was printed using a *Formlabs Form 2 printer* .

## Results

### Raise to Antagonist Cusp Tips

**Fig. 1:**
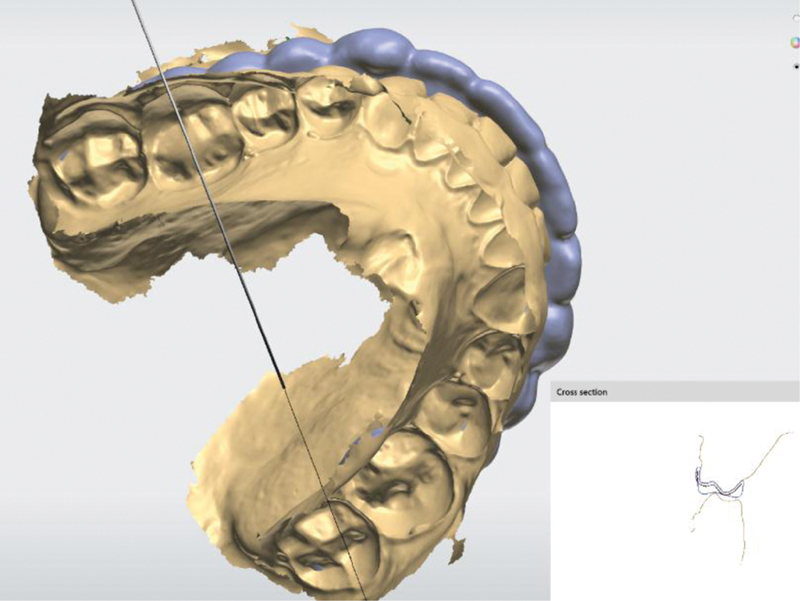
Cross section of the splint raise to antagonist cusp tips type.

RACT is the first option of the menu for a creation of a stabilizing type of splints. The occlusal design corresponds in shape to the antagonists and, when closed, directs the lower jaw to the projected position. There is a slight discomfort at the beginning of treatment from “wandering of the lower jaw,” but after an adaptation period of ∼7 days, patients close in the desired position. The design of the splint is shown in Fig. 1.

### Raise to Antagonist Plane

**Fig. 2:**
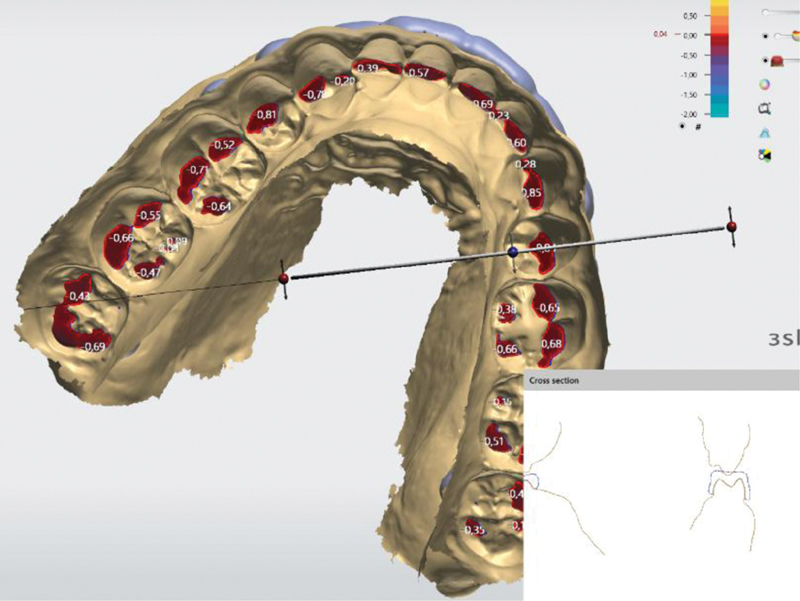
Cross section of the splint raise to antagonist plane type.

This type of splint is characterized by a flat occlusal surface. The digital software fills the central grooves of the distal teeth and generates a flat surface palatal to the front teeth. The splint designed in this way does not fix the position of the lower jaw and allows easy lateral movements. In central occlusion, punctate contact is observed. This splint allows the pathological movements of bruxism and indicates as a better option for orthodontic purposes. The design of the splint is shown in Fig. 2 .

### Raise Ramp

**Fig. 3:**
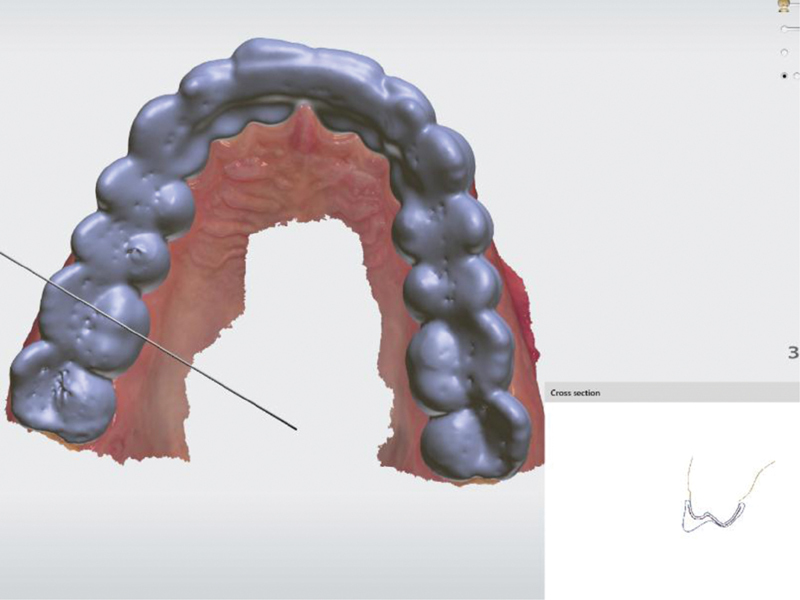
Cross section of the splint raise ramp type.

To create this type of splint, very precise positioning of the occlusal plane is required, which is set as the first step in digital design. This is because the vertical ramps located vestibularly on the splint reach this given occlusal plane. All lateral movements are blocked by these vertical walls. If the entire splint is marked to be made in this way, the front–rear movements are also blocked. The position of the lower jaw is firmly fixed. The profile of the splint is shown in Fig. 3 .

### RACT – Distal + RAP – Frontal

A useful variation in combined design is with occlusion to the antagonists distally and with a horizontal plane frontally. In this way, a fixed position of the lower jaw is achieved, with the possibility of slight lateral movements (it is almost impossible for the patient to suddenly stop performing the pathological friction). The horizontal plane in the frontal area provides contact and prevents the growth of teeth, which is a natural process in the absence of contact.

### RR – Distal + RAP – Frontal

This variation combines vertical ramps distally and a horizontal plane frontally. Lateral movements are completely blocked, and slight anterior–posterior movements are possible, but they are limited by the occlusal relief (although no relief is marked next to the antagonists). The final product is very similar to the design obtained only when using the “RR” type function.

### RAP – Distal + RR – Frontal

This combined splint does not fix the position of the lower jaw, similar to the fully designed RAP splint. The front ramp is positioned differently depending on the occlusion, as it aims to limit forward and backward movements. When positioning the occlusal plane at the level of the incisal edges, it is projected palatally like a horizontal plane and does not perform its complete function. When positioned more than 1 mm higher, it already has a locking function. An increase in VDO is achieved. In nonabrasive tooth surfaces, punctate contacts are observed in the RAP area and more uniform frontally in the RR area. With abraded tooth surfaces, the contacts appear more even.

**Fig. 4:**
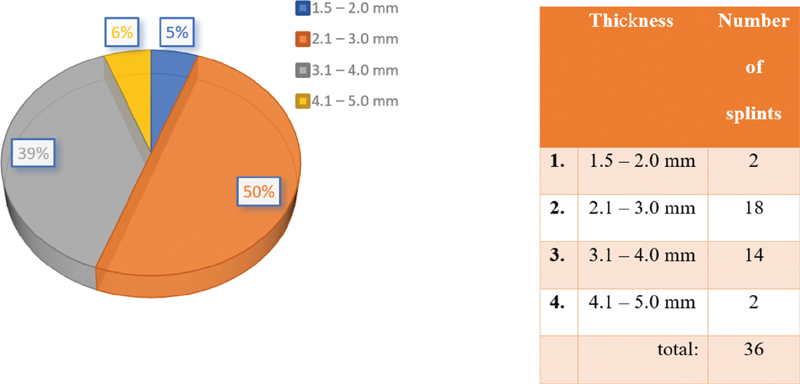
Distribution of the splints by the thickness.

Depending on the thickness of the splints, they can be divided into four groups. The minimum thickness for printing the material is set to 1.5 mm. Occlusal splints over 5 mm are not made by the team ( Fig. 4 ).

## Discussion

The proposed six design options cover most of the known types of splints, classified by different authors—stabilization splint, repositioning splint, anterior bite plane, posterior bite plane, decompression splint, vertical dimension splint, and relaxation splints. Splint type “Raise to antagonist cusp tips (RACT)” combines stabilization splint, repositioning splint, and vertical dimension splint. Splint type “Raise to antagonist plane (RAP)” combines anterior bite plane, posterior bite plane, and vertical dimension splint. The type “Raise ramp (RR)” is repositioning splint, almost fixing. The combined type “RACT − distal + RAP − frontal” represents stabilization splint, repositioning splint and vertical dimension splint. The other combined type “RR − distal + RAP − frontal” can be considered as repositioning splint and anterior bite plane. The last combined type “RAP − distal + RR − frontal” is similar to the posterior bite plane. This proves the universal application of digital software. 9 10 11 12

The boundaries of all splints vary depending on the anatomical features of the teeth and their parallelism to each other. 7 8

The occlusal splints presented in this article do not reach the optimal thickness of 6 mm as recommended by certain authors 14 however, further research to the development of the optimal thickness continues.

## Conclusion

3Shape Digital Design Software—Splint Studio allows the creation of a variety of splints in accordance with the individual needs of the patient. It is possible to combine the three main types in a separate section according to the case. A great advantage of this software is that a digital file is kept on record and more importantly, if further adjustments need to be performed in the future, they can. This allows a continuous development in the types of splints available to the patient.
